# From delay to detection: Dysphagia patterns in tracheostomised ICU patients in South Africa

**DOI:** 10.4102/sajcd.v72i2.1116

**Published:** 2025-11-19

**Authors:** Nancy Barber

**Affiliations:** 1 Department of Speech-Language Pathology, Faculty of Humanities, University of the Witwatersrand, Johannesburg, South Africa

**Keywords:** tracheostomy, transdisciplinary care, dysphagia, speech-language therapy, fibreoptic endoscopic evaluation of swallowing

## Abstract

**Background:**

Dysphagia in tracheostomised patients is highly prevalent but often underdiagnosed, especially in resource-constrained settings such as South Africa. Delays in referral and limited access to instrumental assessments further compromise the quality of care. There is limited data regarding the incidence and severity of dysphagia in patients with tracheostomies within this context.

**Objectives:**

This study aimed to evaluate the incidence of dysphagia in tracheostomised patients at a South African private intensive care unit (ICU) in order to understand and explore the need for context-appropriate, standardised practices of care in this population.

**Method:**

A retrospective file review was conducted on 68 adult patients who underwent fibreoptic endoscopic evaluation of swallowing (FEES) between July 2022 and January 2024. Data were extracted for 42 patients who had a tracheostomy at the time of assessment. Dysphagia severity was rated using the Penetration-Aspiration Scale (PAS). Descriptive statistics were used to analyse incidence, severity, referral timing and ICU stay duration.

**Results:**

A total of 66.6% of tracheostomised patients assessed using FEES presented with dysphagia. Penetration-aspiration scores showed a polarised distribution, with 33.3% scoring 1 (normal) and 28.6% scoring 8 (silent aspiration). The average time to referral was 25.7 days, with FEES completed after 32.4 days. These delays suggest missed opportunities for early identification.

**Conclusion:**

The findings highlight a high incidence of dysphagia and delays in referral, underscoring the need for standardised practices of care to support earlier identification and management.

**Contribution:**

Implementing context-appropriate protocols may improve referral efficiency, optimise resource use and enhance patient outcomes in South African ICUs.

## Introduction

Dysphagia management in South Africa is challenged by systemic pressures, including limited human and material resources, a high burden of trauma-related conditions and the diverse needs of a multilingual, multicultural patient population (Brooke-Sumner et al., 2019). Among critically ill individuals, those with tracheostomies are particularly vulnerable to dysphagia, a complication associated with aspiration, pneumonia, prolonged hospitalisation and increased mortality (Schefold et al., 2017; Zuercher et al., 2019). As breathing and swallowing are neurologically and anatomically interdependent, tracheostomy may compromise the safety and efficiency of swallowing (Skoretz et al., 2020).

Recent data suggest that dysphagia frequently persists post-extubation and post-tracheostomy, often remaining unrecognised until hospital discharge, with up to 93% of tracheostomised intensive care unit (ICU) patients showing signs of disordered swallowing (Likar et al., 2024). Despite this high prevalence, dysphagia is often underdiagnosed and undertreated by speech-language pathologists (SLPs), even in high-risk populations. In South Africa, the implementation of standardised dysphagia practices is further constrained by systemic factors including resource limitations, large caseloads and geographical service disparities (Pillay et al., 2020). These pressures create a service delivery gap that cannot be addressed solely through specialist-driven models. Yet, despite the clear need for feasible and consistent approaches to dysphagia screening, there is a lack of local data that describes the incidence and severity of dysphagia in tracheostomised patients to inform standards of practice. This lack of local data highlights the need to investigate the incidence of dysphagia in tracheostomised patients within a South African private hospital ICU to understand how locally relevant data might support the need to develop and implement context-appropriate, standardised care pathways.

### Literature review

The association between tracheostomy and dysphagia has been extensively documented in other contexts, with prevalence estimates ranging from 11% to 93%, depending on patient population, timing of assessment and method of evaluation (Skoretz et al., 2020). The pathophysiology of ICU-acquired dysphagia is multifactorial and may include direct trauma from artificial airways, impaired sensation, neuromyopathy, poor coordination between respiration and swallowing and critical illness polyneuropathy (Duncan et al., 2020; Zuercher et al., 2019). Dysphagia is an independent predictor of adverse outcomes, including aspiration pneumonia, reintubation, malnutrition and prolonged ICU and hospital stays (Schefold et al., 2017; Skoretz et al., 2020). In many cases, symptoms persist beyond discharge, impacting long-term health and increasing mortality risk (Likar et al., 2024).

Despite this evidence, systematic screening and assessment for dysphagia remain inconsistently implemented across ICU settings. Surveys from the Global North, which broadly refers to high-income, industrialised nations in Europe, North America and parts of East Asia, indicate that fewer than 30% of ICUs use formal dysphagia protocols for tracheostomised patients and fewer than half screen after extubation (Likar et al., 2024; Spronk et al., 2022). In contrast, the Global South comprises lower- and middle-income countries in regions such as sub-Saharan Africa, Latin America and parts of Asia, where resource constraints may further hinder the implementation of standardised screening and intervention protocols (Dados & Connell, 2012). Clinical bedside assessments alone often fail to detect silent aspiration and impaired oropharyngeal clearance (Duncan et al., 2020). Instrumental tools such as fibreoptic endoscopic evaluation of swallowing (FEES) and videofluoroscopic swallowing studies (VFSS) offer improved diagnostic precision but are often unavailable in resource-constrained environments (Langmore, 2017; Langmore et al., 2022).

Given the high prevalence and clinical implications of dysphagia in tracheostomised ICU patients, there is a critical need for standardised practices of care that are evidence based. International expert consensus now recommends clinical algorithms that include systematic screening, timely instrumental assessment and treatment protocols tailored to individual needs (Likar et al., 2024). These algorithms are designed to reduce variability in care, improve patient safety and promote early intervention. Instrumental assessments, particularly FEES, are considered the gold standard for evaluating swallowing function in this population and are vital in guiding appropriate management decisions (Brodsky et al., 2019; Likar et al., 2024). Furthermore, targeted rehabilitative strategies have shown promising outcomes in enhancing swallowing physiology and supporting earlier oral feeding (Bath et al., 2020; Dziewas et al., 2018).

In South Africa, however, resource limitations, large caseloads and geographical service disparities (Pillay et al., 2020) pose challenges to implementing these international recommendations. While algorithm-based screening models are globally advocated (Likar et al., 2024; Skoretz et al., 2020), it remains unclear how such models can be effectively translated to local ICU contexts. Currently, there is a lack of published data on dysphagia prevalence, assessment practices and outcomes for tracheostomised patients within South African hospitals. Without this foundational understanding, it is difficult to determine whether existing global protocols are feasible or effective in local practice. As such, there is a pressing need to evaluate current practices in South Africa to inform the development of standardised practices of care that are both evidence based and contextually relevant.

While evidence for dysphagia interventions in acute and critical care remains limited, some studies have shown that early therapeutic strategies for dysphagia may reduce aspiration pneumonia incidence and facilitate earlier return to oral intake (Duncan et al., 2020; Likar et al., 2024). However, these interventions must be tailored to the patient’s medical stability and the realities of the local healthcare system. Overall, the literature indicates that dysphagia assessment for tracheostomised patients must shift from reactive, specialist-dependent models to proactive, standardised practices of care (Likar et al., 2024). These approaches should be designed to ensure early detection and intervention, reduce avoidable complications and support quality care in both high- and low-resource environments (Likar et al., 2024). Given these gaps, this study aimed to evaluate the incidence of dysphagia in patients with tracheostomies at a private hospital in Johannesburg to understand and explore the need for standardised practices of care in this population.

## Research methods and design

This study aimed to evaluate the incidence and characteristics of dysphagia in adult patients with tracheostomies at a private hospital Johannesburg. A retrospective file review of SLP FEES reports was conducted over 18 months, from July 2022 to January 2024. The study followed a quantitative, non-experimental, descriptive design. This design is appropriate as it enables the measurement of dysphagia incidence without manipulating variables, offering foundational data needed to inform standardised care practices (Brink et al., 2018). This approach is well suited to under-researched clinical contexts where baseline information is lacking.

A purposive convenience sampling approach was used to identify eligible cases. A total of 68 adult patient SLP records were screened. Forty-two SLP records were included for this file review based on the criteria of having undergone a FEES assessment for suspected dysphagia while presenting with a tracheostomy in the critical care unit and from the participating SLP practice. All patient data were anonymised, and each file was assigned a unique code. All patient data were anonymised, and each file was assigned a unique code. Data were securely stored in a password-protected Microsoft Excel© spreadsheet accessible only to the research team. Access will be retained for 5 years in line with ethical data management protocols.

The analysis examined the presence and severity of dysphagia in patients with a tracheostomy. The presence and severity of dysphagia were evaluated from the SLP FEES findings, using the Penetration-Aspiration Scale (PAS), an eight-point ordinal scale designed to categorise the depth of airway invasion and the patient’s response during swallowing. Developed by Rosenbek et al. (1996), the PAS has become a widely used tool in dysphagia assessment. This multidimensional scale captures the depth of invasion, whether the material is ejected and whether the patient demonstrates a response. Its ordinal nature means that while the scores reflect increasing severity, the numerical intervals between scores are not evenly spaced (Borders & Brates, 2020). Table 1 provides the scoring used in the PAS.

**TABLE 1:** The penetration aspiration scale.

Level	Description
1	Material does not enter the airway (normal- no dysphagia)
2	Material does enter the airway, remains above the vocal cords (VC) and is ejected (laryngeal penetration)
3	Material does enter the airway, remains above the vocal cords (VC) and is not ejected (laryngeal penetration)
4	Material does enter the airway, contacts the vocal cords (VC) and is ejected (laryngeal penetration)
5	Material does enter the airway, contacts the vocal cords (VC) and is not ejected (laryngeal penetration)
6	Material does enter the airway, passes through the vocal cords and is ejected from the trachea (aspiration)
7	Material does enter the airway, passes through the vocal cords and is not ejected from the trachea despite effort (aspiration)
8	Material does enter the airway and passes through the vocal cords, and no attempt is made to clear the airway (silent aspiration)

*Source*: Rosenbek, J.C., Robbins, J.A., Roecker, E.B., Coyle, J.L., & Wood, J.L. (1996). A penetration-aspiration scale. *Dysphagia, 11*(2), 93–98. https://doi.org/10.1007/BF00417897

The timing of referrals to SLP was also analysed to identify potential delays in dysphagia management, using the interval between hospital admission and referral as a key indicator.

Descriptive statistical analysis was performed using IBM SPSS Statistics, version 28©, to quantify the incidence and severity of dysphagia and to identify potential patterns in referral timing, providing insight into current practices and highlighting areas where standardised care protocols may be needed or improved.

### Ethical considerations

Ethical approval was obtained from the Human Research Ethics Committee (Medical) at the University of the Witwatersrand (reference number: M240257).

## Results

A total of 68 patient SLP files were reviewed, of which 42 met the inclusion criteria of having a tracheostomy at the time of the FEES. The mean age of these patients was 55.93 years (SD = 17.10), with ages ranging from 25 to 88 years. Table 2 presents descriptive statistics for age, length of stay in the ICU, time to referral to the speech therapist and time to completion of the FEES assessment.

**TABLE 2:** Descriptive statistics for age, time spent in intensive care unit, time to referral to the speech therapist and time to the fibreoptic endoscopic evaluation of swallowing for patients with a tracheostomy (*N* = 42).

Variable	Mean	Median	s.d.	Min	Max
Age (years)	55.93	55.00	17.104	25	88
Time spent in ICU (days)	59.34	42.50	42.305	2	172
Time to referral (days)	25.73	23.50	22.410	2	139
Time to FEES (days)	32.45	32.00	19.840	0	82

ICU, intensive care unit; FEES, fibreoptic endoscopic evaluation of swallowing; s.d., standard deviation; Min, minimum score; Max, maximum score.

These figures show considerable variability in ICU stay and referral timing, suggesting that patients with tracheostomies in this setting often remained in critical care for extended periods before receiving swallowing assessments.

### Dysphagia incidence and severity

Based on the FEES assessment results, 66.6% of the tracheostomised patients had dysphagia. The severity of dysphagia was determined using the PAS, which reflects the degree of airway invasion during swallowing. Table 3 presents the summary statistics for PAS scores.

**TABLE 3:** Penetration-aspiration scale score for fibreoptic endoscopic evaluation of swallowing (*N* = 42).

Variable	Mean	Median	s.d.	Min	Max
PAS score for FEES	4.03	2.00	3.08	1	8

*Note:* The mean PAS score of 4.03 indicates a moderate level of dysphagia on average, with wide variation across the sample. The minimum and maximum scores ranged from 1 (normal swallow) to 8 (silent aspiration), demonstrating that while some patients showed no signs of airway compromise, others presented with severe swallowing impairment.

PAS, penetration-aspiration scale; FEES, fibreoptic endoscopic evaluation of swallowing; s.d., standard deviation; Min, minimum score; Max, maximum score.

Table 4 outlines the frequency and percentage of patients within each PAS score to further describe the severity distribution.

**TABLE 4:** Frequency and percentage of patients within each penetration-aspiration scale score.

PAS score	*n*	%
Score 1	14	33.3
Score 2	8	19.0
Score 3	2	4.8
Score 4	1	2.4
Score 5	1	2.4
Score 6	0	0.0
Score 7	4	9.5
Score 8	12	28.6

PAS, penetration-aspiration scale.

The most frequent PAS scores were 1 (33.3%) and 8 (28.6%), indicating a polarised distribution. While a significant proportion of patients had normal swallowing function, a similarly large group experienced silent aspiration, which poses a high risk of aspiration pneumonia and often goes undetected without instrumental assessment. The remaining patients fell along a continuum of mild to moderate dysphagia severity.

### Referral timing and systemic delays

The data also revealed notable delays in the dysphagia referral process. On average, patients were referred to the speech therapist 25.73 days after admission, and FEES assessments were completed at an average of 32.45 days post-admission. The mean ICU stay was 59.34 days, with some patients remaining in critical care for over 5 months (Table 2).

## Discussion

The findings of this study suggest that dysphagia in tracheostomised patients is not only common but also presents in highly variable forms, ranging from normal swallowing to silent aspiration. This variability reflects the complex and multifactorial nature of dysphagia in critical care, which is influenced by neurological, respiratory and muscular changes associated with prolonged intubation, critical illness and tracheostomy (Schefold et al., 2017; Skoretz et al., 2020; Zuercher et al., 2019). The broad range of presentations underscores the need for standardised practices of care that can sensitively and systematically detect dysphagia, including cases without overt symptoms.

The presence of both low and high PAS scores within the same cohort illustrates the inadequacy of relying solely on clinical observation to identify swallowing impairments. Silent aspiration, in particular, may go undetected in the absence of instrumental tools, placing patients at significant risk for complications such as aspiration pneumonia. While clinical reasoning, bedside assessments and validated screening tools, all contribute to dysphagia identification, their sensitivity remains limited in detecting silent aspiration. The absence or delayed use of instrumental assessments such as FEES may therefore compromise patient safety. These findings reinforce ongoing concerns about the underdiagnosis of dysphagia in intensive care and underscore the importance of embedding structured, objective evaluation methods within standardised care protocols (Duncan et al., 2020; Langmore et al., 2022).

A key finding of this study was the substantial delay in referrals for swallowing assessment. On average, patients were referred to speech-language therapy more than 3 weeks after ICU admission, with FEES occurring even later. These timelines indicate significant inefficiencies in the current referral pathway, where the process is primarily initiated by medical doctors. In the context of a high-acuity ICU, where patients are closely monitored by nursing staff, this doctor-led referral model may hinder early identification – especially in patients with subtle or asymptomatic presentations. This delay may result in extended periods of unmanaged dysphagia, increasing the risk of malnutrition, aspiration and delayed recovery. International literature similarly points to the absence of standardised, multidisciplinary protocols as a contributor to inconsistent referral practices and delayed intervention (Likar et al., 2024; Spronk et al., 2022).

The findings from this study support the need for context-appropriate, standardised care pathways that clearly outline roles, referral triggers and timelines for dysphagia assessment in tracheostomised patients. Such protocols may help to mitigate diagnostic delays by facilitating earlier identification and triage of at-risk patients, particularly if they incorporate clear criteria for when nurses or other ICU staff can prompt or initiate referrals. These improvements would align with international best practices while accounting for resource constraints in the South African context. Moreover, they may enhance interprofessional collaboration without necessitating a full transdisciplinary shift – addressing practical concerns while prioritising patient safety and efficiency of care.

This study contributes to the limited body of evidence on dysphagia in tracheostomised patients from both South African and broader Global South contexts. By providing empirical data on the timing and severity of dysphagia in a private ICU setting, it highlights an important gap between knowledge and practice and identifies opportunities for strengthening clinical systems. However, the study also has limitations. The retrospective design limited access to certain clinical variables and depended on the accuracy and completeness of existing records. Additionally, findings are based on a single hospital site and may not be generalisable to public sector facilities or smaller ICUs with fewer resources.

Future research should explore the design and implementation of locally relevant protocols for dysphagia screening and referral, including the integration of nurse-initiated triggers for assessment. Evaluating the feasibility and effectiveness of such protocols would provide valuable evidence to inform broader policy and practice in South African ICUs. Furthermore, prospective studies are needed to examine how standardised practices of care influence patient outcomes, resource use and interdisciplinary workflows. Furthermore, a replication of this study in the public sector could hold value for future NHI implementation.

## Conclusion

This study highlighted the high incidence of dysphagia among tracheostomised patients in a private ICU setting Johannesburg, South Africa, with many patients experiencing a range of swallowing impairments such as silent aspiration. Despite the clinical significance of dysphagia, the findings revealed considerable delays in referral for assessment. These delays point to inefficiencies in the current referral process, which relies primarily on medical doctors, and suggest that the development and implementation of standard practices of care for this population would be beneficial in improving the timeliness of dysphagia management and optimising resource utilisation. Standard practices of care that empower nurses to participate in early screening could facilitate more proactive identification of at-risk patients, support earlier referrals to speech-language therapists and reduce the incidence of preventable complications. This approach aligns with global best practices and provides a feasible, contextually appropriate strategy for enhancing the quality of care in South African ICUs.

## References

[CIT0001] Borders, J.C., & Brates, D. (2020). Use of the penetration-aspiration Scale in dysphagia research: A systematic review. *Dysphagia*, 35(4), 583–597. 10.1007/s00455-019-10064-3 31538220

[CIT0002] Brink, H., Van Der Walt, C., & Van Rensburg, G. (2018). *Fundamentals of research methodology for healthcare professionals* (4th ed.). Juta.

[CIT0003] Brodsky, M.B., Mayfield, E.B., & Gross, R.D. (2019). Clinical decision making in the ICU: Dysphagia screening, assessment, and treatment. *Seminars in Speech and Language*, 40(3), 170–187. 10.1055/s-0039-1688980 31158902

[CIT0004] Brooke-Sumner, C., Petersen-Williams, P., Kruger, J., Mahomed, H., & Myers, B. (2019). Doing more with less: A qualitative investigation of perceptions of South African health service managers on implementation of health innovations. *Health Policy and Planning*, 34(2), 132–140. 10.1093/heapol/czz017 30863845 PMC6481285

[CIT0005] Dados, N., & Connell, R. (2012). The global south. *Contexts*, 11(1), 12–13. 10.1177/1536504212436479

[CIT0006] Duncan, S., McAuley, D.F., Walshe, M., McGaughey, J., Anand, R., Fallis, R., & Blackwood, B. (2020). Interventions for oropharyngeal dysphagia in acute and critical care: A systematic review and meta-analysis. *Intensive Care Medicine*, 46, 1326–1338. 10.1007/s00134-020-06126-y 32514597 PMC7334257

[CIT0007] Langmore, S.E. (2017). History of fiberoptic endoscopic evaluation of swallowing for evaluation and management of pharyngeal dysphagia: Changes over the years. *Dysphagia*, 32, 27–38. 10.1007/s00455-016-9775-x 28101663

[CIT0008] Langmore, S.E., Scarborough, D.R., Kelchner, L.N., Swigert, N.B., Murray, J., Reece, S., Cavanagh, T., Harrigan, L.C., Scheel, R., Gosa, M.M., & Rule, D.K. (2022). Tutorial on clinical practice for use of the fiberoptic endoscopic evaluation of swallowing procedure with adult populations: Part 1. *American Journal of Speech-Language Pathology*, 31(1), 163–187. 10.1044/2021_AJSLP-20-00348 34818509

[CIT0009] Likar, R., Aroyo, I., Bangert, K., Degen, B., Dziewas, R., Galvan, O., Trapl Grundschober, M., Köstenberger, M., Muhle, P., Schefold, J.C., & Zuercher, P. (2024). Management of swallowing disorders in ICU patients: A multinational expert opinion. *Journal of Critical Care*, 79, 154447. 10.1016/j.jcrc.2023.154447 37924574

[CIT0010] Pillay, M., Tiwari, R., Kathard, H., & Chikte, U. (2020). Sustainable workforce: South African audiologists and speech therapists. *Human Resources for Health*, 18, 1–13. 10.1186/s12960-020-00488-6 32611357 PMC7329495

[CIT0011] Rosenbek, J.C., Robbins, J.A., Roecker, E.B., Coyle, J.L., & Wood, J.L. (1996). A penetration-aspiration scale. *Dysphagia*, 11(2), 93–98. 10.1007/BF00417897 8721066

[CIT0012] Schefold, J.C., Berger, D., Zürcher, P., Lensch, M., Perren, A., Jakob, S.M., Parviainen, I., & Takala, J. (2017). Dysphagia in mechanically ventilated ICU patients (DYnAMICS): A prospective observational trial. *Critical Care Medicine*, 45(12), 2061–2069. 10.1097/CCM.0000000000002765 29023260

[CIT0013] Skoretz, S.A., Riopelle, S.J., Wellman, L., & Dawson, C. (2020). Investigating swallowing and tracheostomy following critical illness: A scoping review. *Critical Care Medicine*, 48(2), e141–e151. 10.1097/CCM.0000000000004098 31939813

[CIT0014] Spronk, P.E., Spronk, L.E., Egerod, I., McGaughey, J., McRae, J., Rose, L., & Brodsky, M.B. (2022). Dysphagia in intensive care evaluation (DICE): An international cross-sectional survey. *Dysphagia*, 37(6), 1451–1460. 10.1007/s00455-021-10389-y 35092486

[CIT0015] Zuercher, P., Moret, C. S., Dziewas, R., & Schefold, J.C. (2019). Dysphagia in the intensive care unit: Epidemiology, mechanisms, and clinical management. *Critical Care*, 23, 1–11. 10.1186/s13054-019-2400-2 30922363 PMC6438038

